# Nitric Oxide (NO) Differentially Modulates the Ascorbate Peroxidase (APX) Isozymes of Sweet Pepper (*Capsicum annuum* L.) Fruits

**DOI:** 10.3390/antiox11040765

**Published:** 2022-04-12

**Authors:** Salvador González-Gordo, Marta Rodríguez-Ruiz, Javier López-Jaramillo, María A. Muñoz-Vargas, José M. Palma, Francisco J. Corpas

**Affiliations:** 1 Group of Antioxidants, Free Radicals and Nitric Oxide in Biotechnology, Food and Agriculture, Department of Biochemistry, Cell and Molecular Biology of Plants, Estación Experimental del Zaidín, Spanish National Research Council (CSIC), Apartado 419, E-18080 Granada, Spain; salvador.gonzalez@eez.csic.es (S.G.-G.); marta.rodriguez@eez.csic.es (M.R.-R.); mangeles.munoz@eez.csic.es (M.A.M.-V.); josemanuel.palma@eez.csic.es (J.M.P.); 2 Institute of Biotechnology, University of Granada, E-18071 Granada, Spain; fjljara@ugr.es

**Keywords:** ascorbate peroxidase, fruit ripening, hydrogen peroxide, nitric oxide, nitration, pepper fruit, peroxynitrite, *S*-nitrosation, ripening, Tyr-nitration

## Abstract

Nitric oxide (NO) is a free radical which modulates protein function and gene expression throughout all stages of plant development. Fruit ripening involves a complex scenario where drastic phenotypical and metabolic changes take place. Pepper fruits are one of the most consumed horticultural products worldwide which, at ripening, undergo crucial phenotypical and biochemical events, with NO and antioxidants being implicated. Based on previous transcriptomic (RNA-Seq), proteomics (iTRAQ), and enzymatic data, this study aimed to identify the ascorbate peroxidase (APX) gene and protein profiles in sweet peppers and to evaluate their potential modulation by NO during fruit ripening. The data show the existence of six *CaAPX* genes (*CaAPX1–CaAPX6*) that encode corresponding APX isozymes distributed in cytosol, plastids, mitochondria, and peroxisomes. The time course expression analysis of these genes showed heterogeneous expression patterns throughout the different ripening stages, and also as a consequence of treatment with NO gas. Additionally, six APX isozymes activities (APX I–APX VI) were identified by non-denaturing PAGE, and they were also differentially modulated during maturation and NO treatment. In vitro analyses of fruit samples in the presence of NO donors, peroxynitrite, and glutathione, showed that CaAPX activity was inhibited, thus suggesting that different posttranslational modifications (PTMs), including *S*-nitrosation, Tyr-nitration, and glutathionylation, respectively, may occur in APX isozymes. In silico analysis of the protein tertiary structure showed that residues Cys32 and Tyr235 were conserved in the six CaAPXs, and are thus likely potential targets for *S*-nitrosation and nitration, respectively. These data highlight the complex mechanisms of the regulation of APX isozymes during the ripening process of sweet pepper fruits and how NO can exert fine control. This information could be useful for postharvest technology; NO regulates H_2_O_2_ levels through the different APX isozymes and, consequently, could modulate the shelf life and nutritional quality of pepper fruits.

## 1. Introduction

Nitric oxide (NO) is a free radical that directly or indirectly exerts signaling functions either through the posttranslational modification (PTM) of proteins, mainly *S*-nitrosation and nitration [1,2,3,4], or by gene regulation [5,6,7,8,9]. In recent years, the interest in NO as a modulator of the postharvest maturation process has grown due to its possible biotechnological applications in maintaining the quality of the fruit, and in avoiding possible infections by pathogens during storage, since it seems that NO induces certain antioxidant systems [2,10,11,12]. Additionally, H_2_O_2_ is an important non-radical reactive oxygen species (ROS) that, at low concentrations, is also involved in plant physiological processes, playing a key role as an active signaling molecule. However, at high concentrations, H_2_O_2_ could be the cause of damage to plants, and thus its concentration should be controlled [13,14].

Ascorbate peroxidase (APX; EC 1.11.1.11) is a heme-containing enzyme that is part of the plant ascorbate–glutathione cycle, which catalyzes the decomposition of H_2_O_2_ according to the following reaction: H_2_O_2_ + L-ascorbate → 2 H_2_O + dehydroascorbate. APX is encoded by a multigene family generating several isozymes whose number differs depending on the plant species, and their expression is regulated according to their tissue/organ distribution, subcellular compartmentalization, stage of growth and development, and environmental conditions [15,16,17,18,19,20,21,22,23]. In *Arabidopsis thaliana*, APXs are encoded by eight genes including three cytosolic (*APX1*, *APX2*, and *APX6*), three peroxisomal (*APX3*, *APX4*, and *APX5*), and two chloroplastic, one at the thylakoidal membrane (*tAPX*) and the other at the stroma (*sAPX*) [20,24,25]. Furthermore, the latter has also been found in the mitochondrion [20,25,26,27]. On the other hand, in tomato (*Solanum lycopersicum*) seven *APX* genes including three cytosolic, two peroxisomal, and two chloroplastic APXs have been reported [28].

Pepper (*Capsicum annuum* L.) fruits belong to the Solanaceae family, together with tomatoes, thus representing a group of horticultural products that are among the most consumed worldwide and, therefore, have great economic and nutritional impact [29,30]. Unlike tomato, which is a climacteric fruit, pepper falls into the category of non-climacteric fruits since its maturation is ethylene-independent. During ripening, pepper fruits undergo nitro-oxidative stress [31,32] where the metabolism of ROS is significantly regulated [6,7,33,34,35,36,37,38].

Considering that APX is a key component in the metabolism of ROS, and based on our previous reports on the transcriptome and proteome of sweet pepper [6,7], this study aimed to identify the APX genes/proteins present in sweet peppers fruits and evaluate if those genes/proteins are modulated during the ripening stage (ripe red vs. immature green), or as a consequence of treatment with NO gas. The obtained data indicated that the enzyme activity, gene, and protein expression of the six identified APXs were differentially regulated during ripening and by NO, and in silico analysis provided evidence for NO-derived post-translational modifications (PTMs) as having potential responsibility for such modulation.

## 2. Materials and Methods

### 2.1. Plant Material and Nitric Oxide Gas Treatment

The criteria for selecting sweet pepper (*Capsicum annuum* L., cv. Melchor) fruits for the experimental plant materials and their subsequent treatments were established according to González-Gordo et al. [6]. Briefly, California-type pepper fruits were collected from plants grown in plastic-covered experimental greenhouses (Syngenta Seeds, Ltd., El Ejido, Almería, Spain). The selected fruits without any external damages at three developmental stages were labeled as green immature (G), breaking point (BP1), and red ripe (R). In the laboratory, the fruits were washed with distilled water and kept for 24 h at a low temperature (about 7 °C ± 1 °C). Then, the selected BP1 fruits were exposed to NO gas treatment (5 ppm NO for 1 h), as reported earlier [39]. Supplementary Figure S1 shows a representative picture of the experimental design followed in this study with the representative phenotypes of sweet pepper fruits at different ripening stages and subjected to NO treatment [6]. The fruits were chosen in this BP1 stage due to the physiological behavior of the ripening of pepper fruits, which are non-climacteric. Thus, the ripening must be initiated (BP1), because if the fruits are harvested in a green stage, the peppers will not ripen to red. This is one of the differences between non-climacteric and climacteric fruits, such as tomatoes, which, if they are harvested in the green stage, later mature to red.

### 2.2. Library Preparation and RNA-Sequencing

Pepper fruit libraries were prepared using an optimized Illumina protocol and were sequenced on an Illumina NextSeq550 platform using 2 × 75 bp paired-end reads [6]. Reads were pre-processed to remove low-quality sequences. With the clean reads, the de novo transcriptome assembly was accomplished. Bowtie2 was used to realign the reads and Samtools was used to quantify the known transcripts. Differential expression analyses were carried out using TransFlow and DEgenes-Hunter, which apply different algorithms with their own statistical tests to validate the whole experiment [6,7] (González-Gordo et al., 2019). Sequence Read Archive (SRA) data are available at the following link https://www.ncbi.nlm.nih.gov/sra/PRJNA668052 (accessed on 28 May 2020).

### 2.3. Proteomic Analysis by iTRAQ^®^ (Isobaric Tags for Relative and Absolute Quantitation)

Preliminary preparation of samples for proteomic analysis was achieved as reported recently [40]. After reduction and alkylation, protein fruit samples were mixed with trypsin at a final trypsin:protein ratio of 1:10 and digested overnight at 37 °C. Tryptic peptides were dried by vacuum centrifugation, reconstituted in labeling buffer (70% ethanol/25 mM TEAB) and labeled with iTRAQ reagents, according to the manufacturer’s protocol (ABSciex, Framingham, MA, USA). Protein identification and quantification were performed by LC-MS/MS analysis using a nanoLC Ultra 1D plus/Triple TOF 5600 analyzer (ABSciex, Framingham, MA, USA).

### 2.4. Phylogenetic and Conserved Motif Analyses of APX Protein Sequences

The alignment of APX proteins was performed using the ClustalW tool with default parameters. A phylogenetic tree using a total of 42 APX protein sequences from *Arabidopsis thaliana*, *Oryza sativa*, *Populus tricocharpa*, *Solanum lycopersicum*, *Solanum tuberosum* and the identified APXs from *Capsicum annuum* was generated using MEGA X [41] and edited with Figtree software. Sequence logos of conserved motifs were created by MEME, Version 5.4.1 [42].

### 2.5. Preparation of Fruit Crude Extracts for APX (EC 1.11.1.11) Activity

Frozen samples of sweet pepper fruits at the different ripening stages and after NO treatment were powdered under liquid nitrogen using an IKA^®^ A11Basic analytical mill (IKA^®^, Staufen, Germany) and then extracted in 100 mM Tris-HCl buffer, pH 8.0, containing 0.1% (*v*/*v*) Triton X-100, 1 mM ethylenediaminetetraacetic acid (EDTA), and 10% (*v*/*v*) glycerol in a final 1:1 (*w*:*v*) plant material: buffer ratio. Homogenizing solutions for crude extracts prepared to measure APX activity also contained either 2 mM (spectrophotometric determination) or 5 mM (isoenzyme detection in acrylamide gels) ascorbic acid. The obtained homogenates were centrifuged at 15,000× *g* for 30 min at 4 °C and the supernatants were used for enzymatic APX assays. APX was determined spectrophotometrically by monitoring the initial ascorbate oxidation by H_2_O_2_ at 290 nm [43]. To study the modulation of APX activity, in vitro incubations of crude extracts from sweet pepper fruit in the presence of 3-morpholinosydnonimine (SIN-1, 0.1–1 mM, a peroxynitrite (ONOO^−^) donor), *S*-nitrosoglutathione (GSNO, 0.5–2 mM, an NO donor), and reduced glutathione (GSH, 0.5–2 mM) were carried out, and then the spectrophotometric assay was performed [44].

### 2.6. In-Gel Isozyme Profile of APX Activity and In Vitro Treatments

For the detection of APX in gels, the method reported by Bieker et al., (2012) [45] with some modifications was followed [46]. Non-denaturing PAGE was carried out in 10% acrylamide gels (19:1, acrylamide:bis-acrylamide ratio) using a Mini-Protean Tetra Cell (Bio-Rad, Hercules, CA, USA). The native-PAGE was run with a standard running buffer containing 2 mM ascorbate. Pepper fruit crude extracts were added in addition to 0.006% (*w*/*v*) bromophenol blue dye and then loaded onto gels. Pre-electrophoresis was initially run at 15 mA/gel for 30 min, and then the current was set at 25 mA/gel until the dye front reached 1 cm above the gel edge. After electrophoresis, the gels were first incubated in 50 mM K-phosphate buffer, pH 7.0, containing 2 mM ascorbic acid for 30 min, and thereafter in the same buffer containing 4 mM ascorbic acid plus 0.5 µM H_2_O_2_ for 10 min. Then, gels were equilibrated with 50 mM K-phosphate pH 7.8 for 1 min. Finally, the gels were incubated in 50 mM K-phosphate buffer, pH 7.8, containing 12 mM TEMED and 2 mM NBT (nitroblue tetrazolium). APX activity was observed as an achromatic band on a purple–blue background and the staining was stopped by rising the gels with water.

For the APX assays after in vitro treatment, fruit samples were pre-incubated at 25 °C for 1 h with different compounds, including *S*-nitrosocysteine (CysNO, 5 mM) as the NO donor [35,47,48], and dithiothreitol (DTT, 5 mM), GSH (0–5 mM), and L-cysteine (L-Cys, 5 mM) as the reducing agents. Then, they were subjected to electrophoresis. Likewise, the treatment with SIN-1 was performed at 37 °C for 1 h [49,50]. Additionally, to determine the thermal stability of APX activity, fruit samples were also pre-incubated at 95 °C for 5 min before electrophoresis.

### 2.7. Protein Modeling

APX sequences were submitted to the M4T [51], Phyre2 [52], RaptorX [53], and Swiss-model [54] servers. No restriction was imposed on the selection of the template. Since each server implements a different algorithm (i.e., force field, search strategy, and score function), results were further evaluated with the SAVES 6.0 server in terms of non-bonded atom–atom interactions (ERRAT) [55], compatibility among the atomic model and the amino acid sequence (VERYFY 3D) [56], stereochemical quality (PROCHECK) [57], and the global score QMean4 [58]. Models were inspected with UCSF Chimera [59].

### 2.8. Other Assays

Protein concentration was determined using the Bio-Rad protein assay (Hercules, CA, USA), with bovine serum albumin as standard. The intensity of the bands identified in the gels was quantified using ImageJ 1.45 software (https://imagej.nih.gov/ij/, accessed on 3 December 2021).

## 3. Results

### 3.1. APX Genes/Proteins from Sweet Pepper Fruits: Sequence, Structure, and Phylogenetic Analysis

APX is a heme peroxidase that constitutes a key enzyme in the metabolism of H_2_O_2_ produced in different subcellular compartments. To obtain a deeper knowledge of the APX isozymes present in pepper fruit and how they could be modulated during ripening, the identification of the APX genes was carried out based on the sweet pepper transcriptome previously reported [6], and their corresponding protein sequences were further deduced. Thus, considering the information available on APX, we identified a total of six *APX* genes that were designated as *CaAPX1* to *CaAPX6* according to their distribution in the pepper genome [60]. Table 1 summarizes some of the basic information about the nucleotide and amino acid sequences of the six APXs including their subcellular location. Although it may appear evident, the APXs were clearly identified because these enzymes contain two characteristic motifs: the heme and the ascorbate-binding sites [61,62]. This analysis allowed us to distinguish other APX homologs that were designated as APX-related (APX-R) (data not shown) due to their display of ascorbate-independent heme peroxidase activity [63,64].

**Figure 1:**
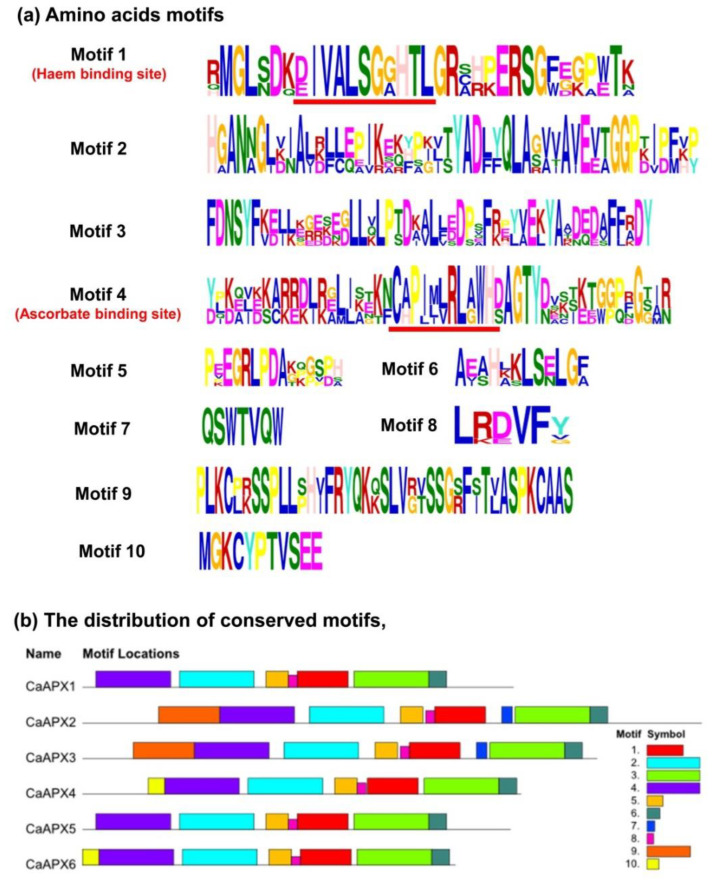
Identification and position of consensus amino acid motifs for sweet pepper APX isozymes. (**a**) Ten amino acid motifs with various widths were identified including the haem and ascorbate binding sites, motifs 1 and 4, respectively. The height of each amino acid symbol is proportional to the degree of conservation in the consensus sequences depicted in the ten motifs. (**b**) The distribution of conserved motifs, numbers 1–10, of the six sweet pepper APX proteins, are represented by boxes of different colors. Sequence logos of conserved motifs were created by MEME.

The chromosomal locations of *CaAPX* genes were physically mapped on the 12 chromosomes of *C. annuum*, but they were distributed on chromosomes 2, 4, 6, 8, and 9. Thus, chromosome 2 contained *CaAPX1*, which encoded the peroxisomal isozyme; chromosome 4 contained *CaAPX2,* which encoded a plastidial/mitochondrial isozyme; chromosome 6 harbored *CaAPX3* and *CaAPX4*, and coded for a second plastidial/mitochondrial isozyme and a cytosolic one, respectively; chromosome 8 hosted *CaAPX5*, encoding a second peroxisomal isozyme; and chromosome 9 contained *CaAPX6* which coded for a second cytosolic isozyme. The analysis of the protein sequences of these six CaAPX isozymes allowed discrimination until the tenth motif, with the motifs 1 and 4 corresponding to the heme and ascorbate binding sites, respectively (Figure 1a). The distribution of these ten conserved motifs in the six sweet pepper APX proteins is represented in Figure 1b.

**Figure 2:**
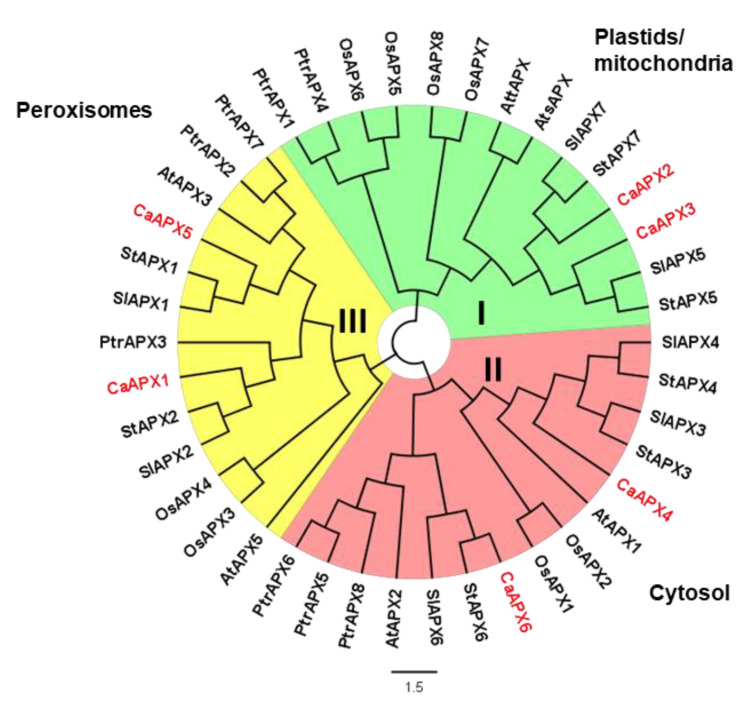
Phylogenetic analysis of CaAPX proteins from diverse plant species. The evolutionary history was inferred by using the Maximum Likelihood method and JTT matrix-based model. The tree with the highest log-likelihood is shown. This analysis involves 42 amino acid sequences. Evolutionary analyses were conducted in MEGA X. The scale bar represents the phylogenetic branch length. Clade I, II, and III include the chloroplastic/mitochondrial, cytosolic, and peroxisomal APX proteins, depicted in green, red, and yellow, respectively. Species abbreviations: At (*Arabidopsis thaliana*); Ca (*Capsicum annuum*); Os (*Oryza sativa*); Ptr (*Populus trichocarpa*); Sl (*Solanum lycopersicum*); and St (*Solanum tuberosum*).

The analysis of the primary structure of the six CaAPX isozymes revealed a high degree of identity between CaAPX1 and CaAPX5 (85%), CaAPX2 and CaAPX3 (92%), and CaAPX4 and CaAPX6 (87%) (Supplementary Table S1). A phylogenetic comparative analysis among the APX proteins from six plant species including sweet pepper (*Capsicum annuum*), *Arabidopsis thaliana*, rice (*Oryza sativa*), populus (*Populus trichocarpa*), tomato (*Solanum lycopersicum*), and potato (*Solanum tuberosum*) allowed the identification of three main APX groups, designated as I to III and depicted with different colors in Figure 2. Group I represents plastidial and mitochondrial isozymes, and includes CaAPX2 and CaAPX3. Group II resembles cytosolic APX isozymes and encloses CaAPX4 and CaAPX6. Group III corresponds to peroxisomal APXs and includes CaAPX1 and CaAPX5.

### 3.2. NO gas Differentially Modulates APX Genes and Protein Expression during Ripening

**Figure 3:**
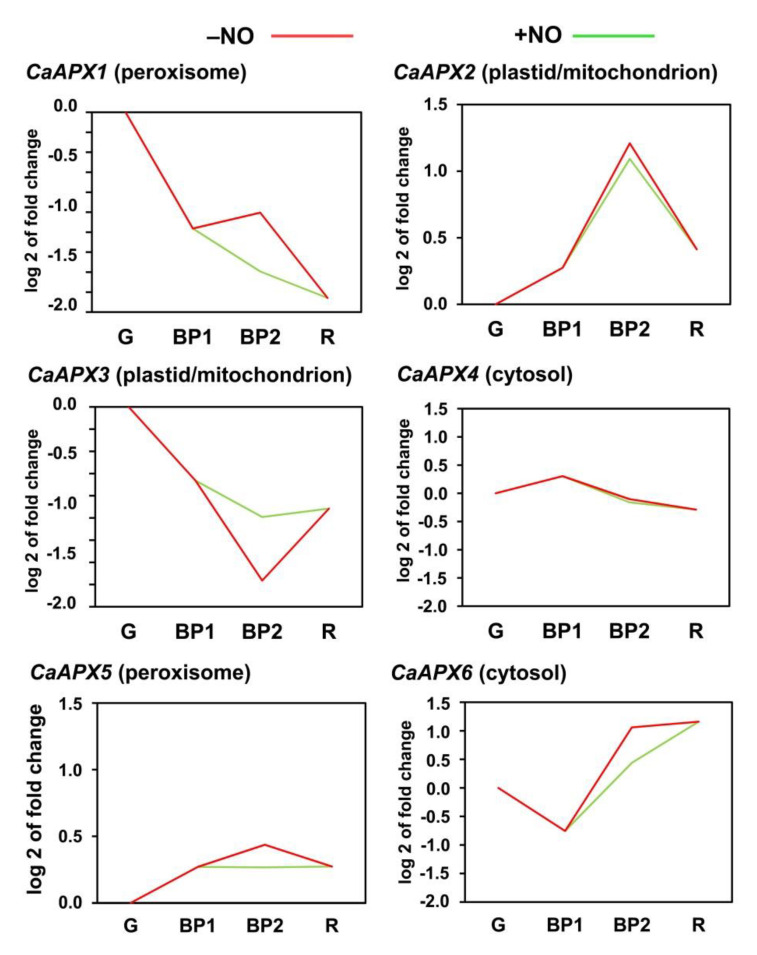
Time course expression analysis of six *CaAPX* genes (RNA-Seq). Samples of sweet pepper fruits at different ripening stages correspond to immature green (G), breaking point 1 (BP1), breaking point 2 with (green line) and without (red line) NO treatment (BP2 + NO and BP2 − NO, respectively) and ripe red (R).

Once the six *CaAPX* genes were identified in the sweet pepper fruits, we analyzed how their expression was modulated during ripening and the potential effect of NO gas treatment in comparison to untreated fruits. Figure 3 displays the time course expression analysis of these six *CaAPX* genes by RNA-Seq at different stages of pepper fruit ripening. In general, we observed a dissimilar response to the different *CaAPX* genes which encoded the six CaAPX isozymes present in diverse organelles. Thus, regarding the genes encoding the peroxisomal isozymes it was found that, while *CaAPX1* was downregulated from the green (G) to red (R) stages, *CaAPX5* was upregulated. With respect to NO treatment (BP2 + NO vs. BP2 − NO), *CaAPX1* was downregulated by NO (green line) in comparison to the untreated fruits (red line), and *CaAPX5* was also slightly downregulated. In the case of the genes that coded for the plastid and mitochondrion isozymes, it was observed that, during ripening (R versus G), *CaAPX2* was upregulated and *CaAPX3* was downregulated. However, the NO effect in the BP2 stage caused an increase in *CaAPX3* expression but a slight decrease in *CaAPX2*. Finally, in the genes that encoded the cytosolic isozymes, *CaAPX4* underwent a slight decrease during ripening and was unaffected by NO, whereas the expression of *CaAPX6* was upregulated by ripening and the NO treatment slightly decreased its expression at the BP2 stage, in comparison to the untreated fruits.

**Figure 4:**
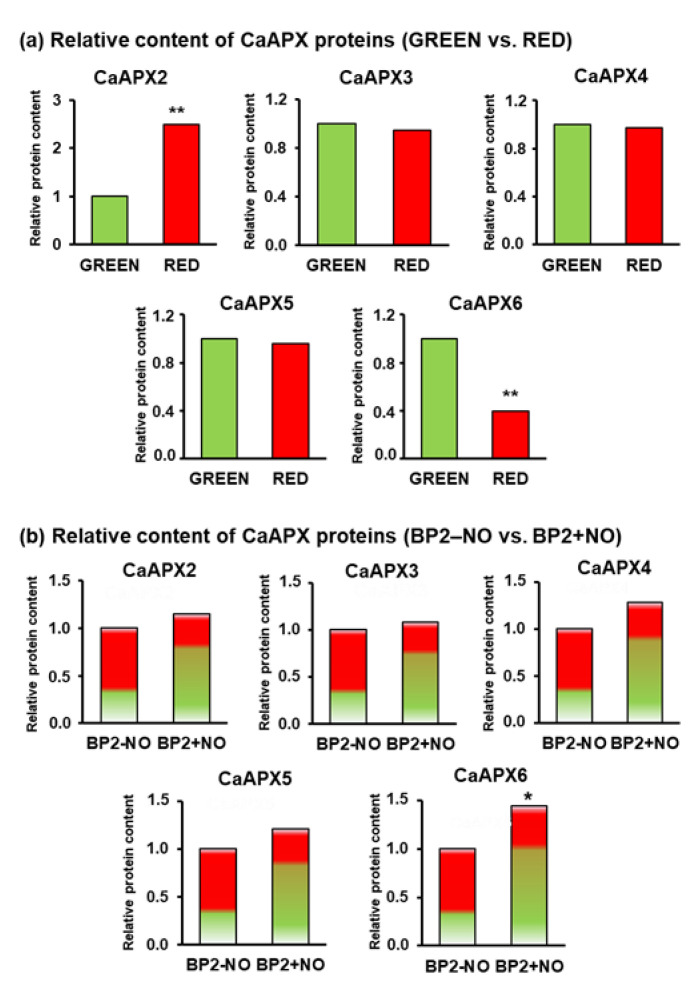
Time course protein expression analysis of five CaAPX isozymes identified by iTRAQ. (**a**) Comparative protein expression of CaAPX isozymes between pepper fruit at green and ripe red stages. (**b**) Comparative protein expression of CaAPX isozymes of pepper fruits, at breaking points, treated or untreated with NO. Samples of sweet pepper fruits at different ripening stages correspond to immature green, ripe red, and breaking point 2 with and without NO treatment (BP2 + NO and BP2 − NO, respectively). Asterisks indicate that differences between values were statistically significant at *p* < 0.05.

The protein expression analysis was performed using the proteomes obtained by iTRAQ at each stage of fruit ripening. In the proteome, it was possible to identify only five CaAPX isozymes since the peroxisomal CaAPX1 was not detected. A comparative analysis was made by comparing how the protein expression of these APXs changed by ripening, that is, considering green and red fruits. Figure 4a illustrates that the expression of CaAPX2 increased around 2.4-fold in ripe red fruits, CaAPX6 decreased about 3-fold and, by contrast, the protein expression of CaAPX3, CaAPX4, and CaAPX5 did not undergo significant changes. On the other hand, a comparative analysis of the expression of five isozymes after the NO treatment was also performed. Figure 4b shows that only the expression of cytosolic CaAPX6 underwent an increase of around 45% due to the NO treatment, while the protein expression of the other CaAPX isozymes did not experience significant changes.

### 3.3. APX Isozyme Profile in Sweet Pepper Fruits

**Figure 5:**
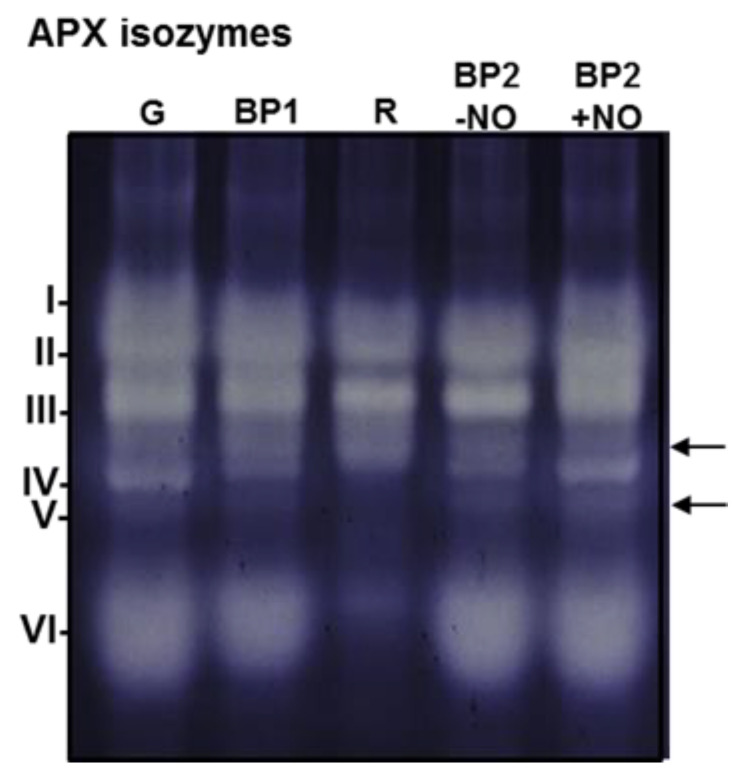
Ascorbate peroxidase (APX) isozymes in samples of sweet pepper fruits at different stages of ripening. Samples correspond to immature green (G), breaking point 1 (BP1), ripe red (R), and breaking point 2 with and without NO treatment (BP2 + NO and BP2 − NO, respectively). Protein samples (30 µg per lane) were separated by non-denaturing polyacrylamide gel electrophoresis (PAGE; 10% acrylamide), and activity was detected by the nitro blue tetrazolium (NBT)-reducing method. APX isozymes were labeled I–VI (on the left) according to their increasing electrophoretic mobility. Arrows indicate some minority bands.

The biochemical characterization of the CaAPX isozymes by non-denaturing PAGE during fruit ripening and under NO treatment was also accomplished. Figure 5 illustrates the presence, in green fruits, of a total of six CaAPX isozymes designated to APX I to APX VI, according to their increasing electrophoretic mobility. The activity intensity of some of these isozymes was modulated during ripening and by NO effects. Overall, the intensity of some bands diminished significantly, which is in good agreement with our previous data, where the total APX activity assayed spectrophotometrically decreased 2.5-fold in red fruits [6]. The nomenclature attributed to the APX isozymes identified in the non-denaturing gels by PAGE was different from the previous one because it was not possible to establish a direct correlation between them and the CaAPX proteins detected by iTRAQ. Making such a correlation would require the purification of each of the isozymes and subsequent analysis by native PAGE. Among the six CaAPXs detected in the gels, APX II, III, and VI were the most prominent bands in green fruits, whereas in red fruits APX IV and V diminished or even disappeared. Likewise, a new band above the APX IV isozyme was detected in both ripe and BP stages, but not in green fruits (see arrow in Figure 5). At the same time, APX VI, which appeared to be most prominent in green fruits, also lowered drastically in red fruits. Overall, it is remarkable that, except APX I, most APX activity bands were modulated either during ripening or by the NO gas treatment.

**Figure 6:**
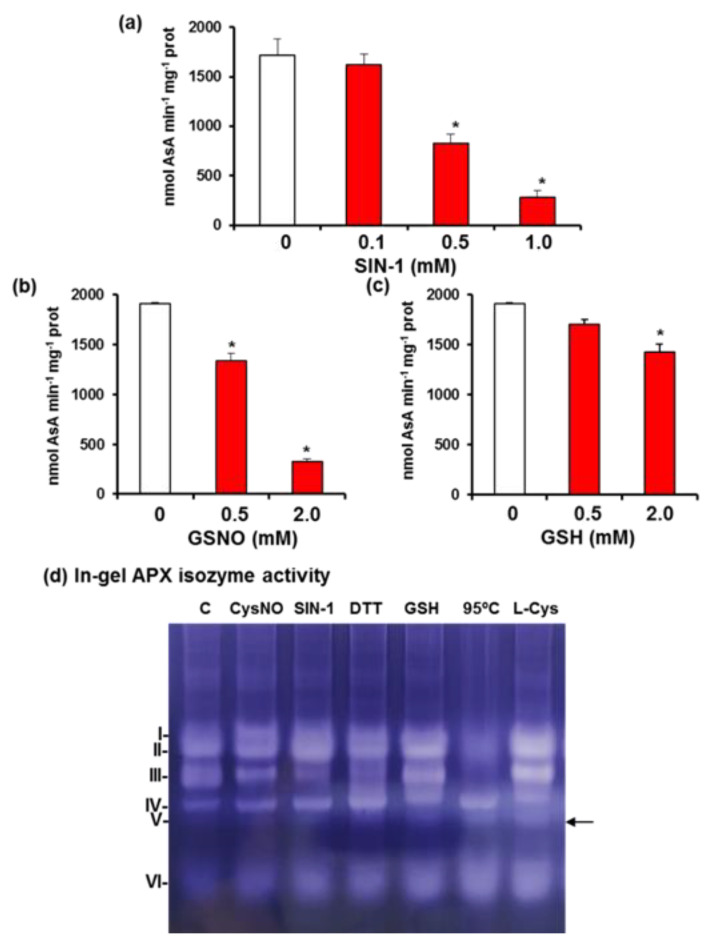
Effect of nitration, *S*-nitrosation and reducing agents on isoenzymatic ascorbate peroxidase (APX) activity in green pepper fruits. (**a**) Effect of SIN-1 (peroxynitrite donor). (**b**) Effect of *S*-nitrosoglutathione (GSNO). (**c**) Effect of reduced glutathione (GSH). (**d**) In-gel isozyme profile of APX activity assay under different treatments including 5 mM nitrosocysteine (CysNO, a NO donor), 5 mM SIN-1, 5 mM DTT, 95 °C for 5 min, and 5 mM L-cysteine (L-Cys). Protein samples (30 µg per lane) were separated by non-denaturing polyacrylamide gel electrophoresis (PAGE; 10% acrylamide), and activity was detected by the nitro blue tetrazolium (NBT)-reducing method. APX isozymes were labeled I–VI (on the left) according to their increasing electrophoretic mobility. Spectrophotometric APX activity data represent the mean + SEM of at least three independent biological replicates. Asterisks (*) indicate significant differences (*p* < 0.05) in comparison to the control. Arrow indicates an induced band (CaAPX V) not detected in the control sample.

In previous studies, we have shown that pepper fruits undergo nitro-oxidative stress since the metabolism of ROS and reactive nitrogen species (RNS) are significantly up-modulated. Therefore, to obtain deeper knowledge on the CaAPX activity, we carried out in vitro assays using different compounds involved in the RNS metabolism, including SIN-1, a peroxynitrite (ONOO^−^) donor and nitrating reagent, *S*-nitrosoglutathione (GSNO) and nitrosocysteine (CysNO) as NO donors, and the reducing compounds GSH, L-Cys, and DTT, both by spectrophotometric assay (Figure 6a–c), as well as the in-gel APX activity assay (Figure 6d). Figure 6a shows that the total APX activity in the presence of an increasing SIN-1 concentration underwent a progressive inhibition that was around 67% with 1 mM of SIN-1. A similar effect was observed with GSNO which, at 2 mM, caused an 80% inhibition (Figure 6b). GSNO decomposes into GSH and NO, so the effect of GSH was also analyzed (Figure 6c), and it was shown that GSH also had an inhibitory effect of about 30%. Figure 6d illustrates the in-gel APX isozymatic analysis in the presence of several compounds. Thus, CysNO increased the band intensity of APX I, whereas it diminished APX III. SIN-1 lowered the APX III, but enhanced APX II and IV. DTT also diminished APX III, but GSH stimulated this isoenzyme, as well as APX IV, V, and VI, slightly. On the other hand, L-Cys increased the band intensity of APX V and VI. Finally, to evaluate the stability of these activities, the fruit samples were heated at 95 °C for 5 min previous to electrophoresis and, surprisingly, APX IV, V, and VI were unaffected.

### 3.4. Modeling of the Tertiary Structure of the Six CaAPX Isozymes

To gain additional insight into the different behavior of the CaAPX isozymes, and considering that the structures of different APXs are available at PDB, they were modeled using the servers M4T, Phyre2, RaptorX, and the Swiss model. Since these servers implement different algorithms, the best results according to the score function of each server were further evaluated in terms of non-bonded atom–atom interactions [55], compatibility among the atomic model and the amino acid sequence, stereochemical quality, and the global score QMean4. In general, the four servers succeeded in the modeling of the six CaAPX isozymes, although those output by Phyre2 and the Swiss model showed the best scores during the evaluation (see Supplementary Tables S2–S7).

The final CaAPX models were the coordinates obtained from the Swiss model, except for plastid/mitochondrion APX3, which were from Phyrex2 (Supplementary Table S8). The analysis of the models revealed that the folding was well preserved and that the resulting structures were superimposable, with a normalized RMS (root mean square) value lower than 1.0 Å (Supplementary Table S9). CaAPX1 and CaAPX5, sharing 85% amino acid identity, and whose phylogenetic analysis placed them within Group III—corresponding to peroxisomal APXs—are structurally quite similar, with an RMS of 0.073 Å. The same applies for plastidial/mitochondrial APX2/APX3 and cytosolic APX4/APX6, with RMSs of 0.260 Å and 0.252 Å, respectively.

**Figure 7:**
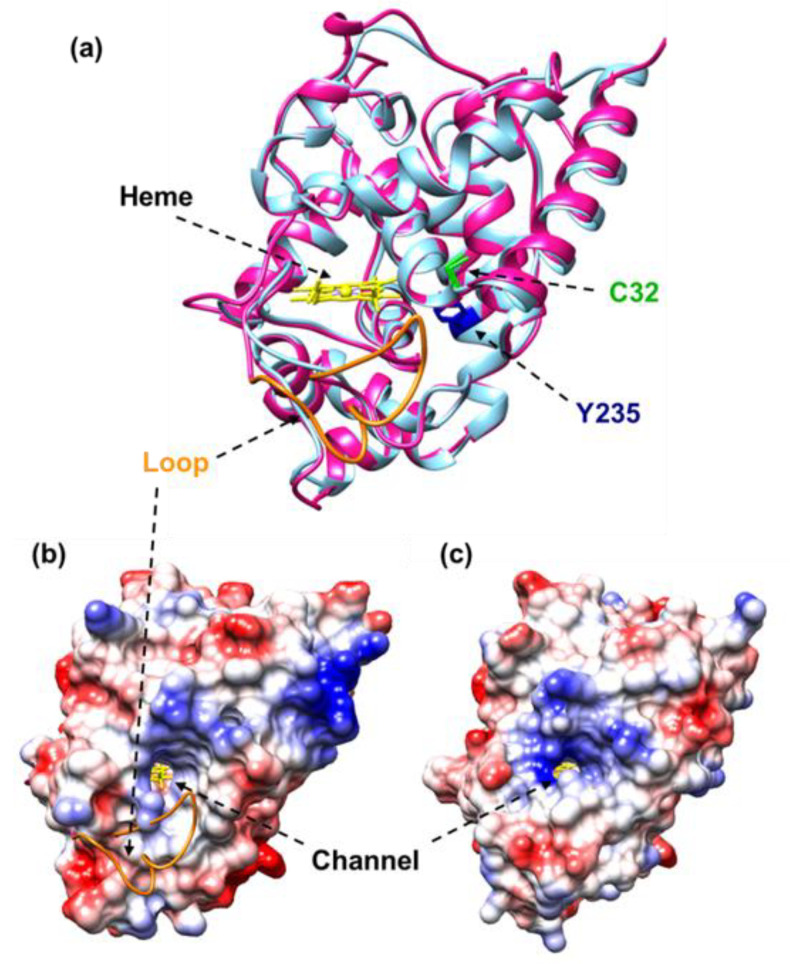
Comparative analysis of the models of plastidial/mitochondrial CaAPX3 and cytosolic CaAPX4. (**a**) Superposition of CaAPX3 (magenta) on CaAPX4 (cyan) showing the extra loop of CaAPX3 in orange, the heme group in yellow, and residues that undergo NO-derived PTMs (C32 in green and Y235 in blue). (**b**) The surface of CaAPX4 shows the position of the CaAPX3 loop (orange) and the access channels connecting the surface with the distal heme pocket. (**c**) The surface of CaAPX3 in the same orientation illustrates how the loop interferes with the orientation of the channel.

**Figure 8:**
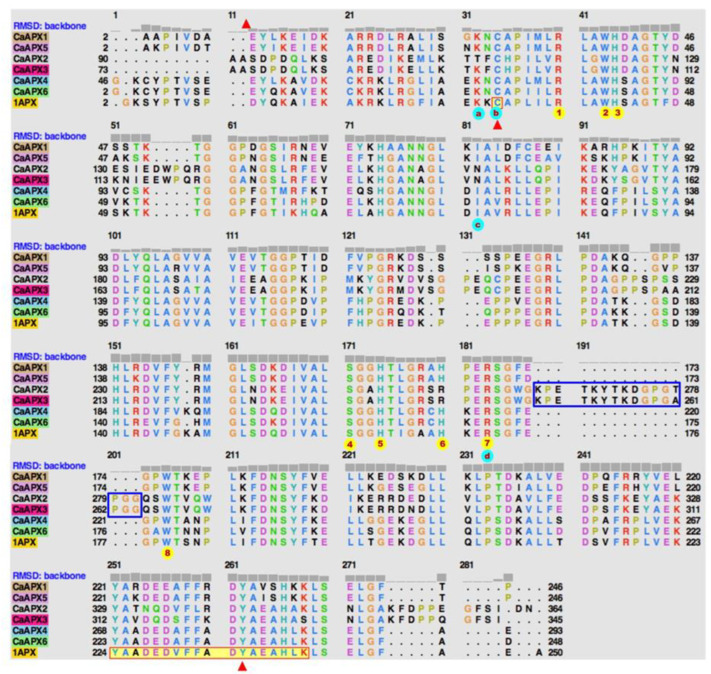
Alignment of the primary structure of the six CaAPX isozymes and the pea cytosolic APX (1APX) resulting from the superimposition of the coordinates of CaAPX isozymes on the structure of 1APX. Residues comprising the active site of 1APX (R38, W41, H42, S160, H163, H169, R172, and W179) are denoted by a number in red encircled in yellow. Residues involved in the interaction with ascorbic acid (K30, C52, I76, R172) are denoted by a letter in red encircled in blue. Residues reported to undergo NO-derived PTMs are highlighted by a red solid triangle and the peptide identified by MS/MS is shown on a yellow background. The loops present in CaAPX2 and CaAPX3 are enclosed in blue rectangles.

Previous studies on the modulation of the cytosolic pea APX have demonstrated that it undergoes a dual regulation; it is inhibited by peroxynitrite and enhanced by GSNO, with Y235 and C32 as the residues that undergo the corresponding NO-mediated posttranslational modifications (PTMs) of nitration and *S*-nitrosation, respectively [44]. In this context, the models of the CaAPX isozymes were further analyzed by the superposition of the coordinates on the structure of pea APX (PBD accession code 1APX) (Figure 7a–c). As expected, the models fit on 1APX with a root mean square deviation (RMSD) lower than 1 Å, structural distance measure (SDM) scores [65] ranging from 6.7 to 19.6, and a Q score [66] of 0.74 for the plastidial CaAPX2 and CaAPX3, and higher than 0.96 for the other CaAPX isozymes (Supplementary Table S10). At this point, it is important to recall that SDM is zero for identical structures and increases with dissimilarity, and that Q scores range from zero for no superimposable structures to 1 for identical structures. Hence, RMSD, SDM, and Q scores indicate that the CaAPX isozymes fit very well on pea 1APX, and the matched residues are depicted in Figure 8**.** Both C32 and Y235 are preserved in the six CaAPX isozymes. Additionally, the residues of R38, W41, H42, S160, H163, R172, and W197 of pea 1APX, involved in the active site [62], are absolutely preserved, and H169 is replaced by R in the plastidial/mitochondrial CaAPX2 and CaAPX3. Residues involved in the interaction with the ascorbate are K30 and C32, although they play a minor role, and I76, which lies on top of the ascorbate and contributes to maintaining the position for the H-bonding with R172, is essential for the catalysis [62,67]. Interestingly, I72 is not preserved in the plastidial/mitochondrial CaAPX2 and CaAPX3; however, it presents an extra loop comprising position 188–203 (numbering of the alignment is shown in Figure 8).

## 4. Discussion

H_2_O_2_ is part of the ROS metabolism and under physiological conditions exerts signaling function [68,69]. Plants have a diverse battery of enzymatic antioxidant systems to keep H_2_O_2_ content under control in different subcellular compartments, and APX is one of the key enzymes that achieves this function [18]. Sweet pepper fruits are highly consumed worldwide and have diverse culinary uses, thus promoting beneficial properties for human health [70]. At a physiological level, pepper fruit ripening is linked to many phenotypical changes, its color shift being one of the most obvious. In previous reports, we have shown that, during ripening, sweet pepper fruits undergo nitro-oxidative stress [31,32,33], characterized by changes in some components of NO metabolism such as *S*-nitrothiols content, GSNO reductase activity, protein tyrosine nitration [31,37,71,72], and the modulation of the NADPH-generating system and antioxidant enzymes [49,50,73]. This includes catalase [36,38], as well as enzymes involved in the metabolism of superoxide radicals, including NADPH oxidase and superoxide dismutase (SOD) isozymes [7,35]. Furthermore, it has been demonstrated that the application of exogenous NO gas to pepper fruits provides the capacity to delay the ripening process and to provoke a 40% increase in ascorbate content by affecting the gene expression and activity of galactono-1,4-lactone dehydrogenase [37].

Both H_2_O_2_ and NO are candidates to be second messengers during plant responses mediated by ROS/RNS, due to their relative long life, lower damage capacity, and higher permeability across biological membranes. Physiological processes such as seed germination, stomatal closure, fruit ripening, and plant responses against abiotic and biotic stresses, among others, are regulated by both molecules [74,75,76]. To obtain a deeper knowledge of the implication of ROS and NO metabolisms and their crosstalk during the ripening of sweet pepper fruits, the goal of this study was to focus on the implication of APX as the key enzyme system that regulates the H_2_O_2_ content in different subcellular compartments, and its modulation by NO.

The obtained data indicated that, during the ripening of pepper fruits, some out of the six CaAPX isozymes identified underwent a complex regulation of gene and protein expression levels, in addition to their activity levels, resulting in the downregulation of the APX V and APX VI isozymes that were undetectable in the red stage. Furthermore, the exogenous application of NO gas also exerted a positive effect on APX IV. This is in good agreement with some previous studies where recombinant APXs from leaves of pea and Arabidopsis underwent either an increase in activity due to *S*-nitrosation or an inhibition by nitration [44,77,78,79]. In this sense, the pharmacological analyses using different NO and ONOO^−^ donors corroborate that the total APX activity from pepper fruits is susceptible to be regulated by both PTMs.

On the other hand, the in vitro analysis of the CaAPX isozymes by non-denaturing gels revealed how their activity might be dissimilarly modulated by different biological compounds (CysNO, GSH, L-Cys) in a cellular context that, in most cases, is different than the analyses performed using a single recombinant APX protein. It could be suggested that NO can modulate the level of H_2_O_2_ by regulating, to some extent, the activity of APX isozymes present in the cytosol, mitochondria, plastids, and peroxisomes, as these are mechanisms of crosstalk between these two signal molecules [80]. This NO regulation by PTMs has been described for other antioxidant enzymes [81], such as catalase [36,38], superoxide dismutase [82], and monodehydroascorbate reductase [83], as well as for the redox state of the cell through the regulation of diverse NADPH-generating dehydrogenases [49,50,84,85,86]. Furthermore, NO also negatively regulates superoxide-generating NADPH oxidase [7,35,87], which finally affects the dismutation of superoxide radicals to H_2_O_2_. Another factor that must be considered, and that might explain the differences observed between isolated recombinant proteins and those analyzed in complex plant extracts, is that NO is generated in different subcellular compartments [88], so its interaction with specific antioxidant enzyme systems will depend on other factors, such as the endogenous pH, redox state of a specific organelle, and the relative abundance of both NO and the potential target protein.

From a structural point of view, the six CaAPXs share a common folding. The presence of the residues, reported as targets of the PTMs that yield the regulation by peroxynitrite (nitration) and by GSNO (*S*-nitrosation), suggest a similar dual regulation [44]. However, some subtle changes are present in the plastidial/mitochondrial CaAPX2 and CaAPX3, including the presence of a loop comprising residues 188–203 (numbering of the alignment is shown in Figure 8), and the replacement of the I72 and H169 by Arg and Asn, respectively, two residues in which pea 1APX is involved in the interaction with the heme group and with the ascorbic acid-binding. The loop is located in the vicinity of the propionate side chain of the heme, and one of the two access channels connecting the surface with the distal heme pocket is positioned obliquely instead of horizontally, as in the other isozymes (Figure 7). These features resemble the chloroplastic APX from tobacco plants and support the chloroplastic location of APX2 and APX3 and augur enzyme instability in the absence of ascorbic acid, a common characteristic of chloroplastic APX isozymes, and an eventuality that has been justified as a consequence of a movable heme that becomes degraded (Wada et al., 2003). Furthermore, these changes could also explain the observed differences of the different CaAPX genes compared to the in vitro-tested compounds (Figure 6a).

## 5. Conclusions

APX, as part of the ascorbate–glutathione cycle, is responsible for controlling the levels of H_2_O_2_ in the different subcellular compartments (cytosol, plastids, mitochondria, and peroxisomes) where different isozymes are located. Thus, each specific activity, as well as its gene and protein expression, must be coordinated with each other, as well as with other antioxidant enzymes such as catalase, to break down H_2_O_2_. To our knowledge, the present study provides the first instance of information on APXs from sweet peppers during fruit ripening exposed to an enriched NO atmosphere. This study therefore provides a wide landscape on gene and protein expression using high throughput sequencing approaches (RNAseq and iTRAQ, respectively), plus isoenzymatic activity profiles. Thus, six *CaAPX* genes were identified, encoding six isozymes present in cytosol (CaAPX4 and CaAPX 6), plastids/mitochondria (CaAPX2 and CaAPX 3), and peroxisomes (CaAPX1 and CaAPX 5) whose expression was modulated by both ripening and NO treatment. In vitro analyses using different molecules involved in NO metabolism also indicate the complex mechanisms of activity regulation. Some isozymes underwent nitration (APX III) and *S*-nitrosation and experienced increased APX I activity, whereas the activity of APX III was diminished, and others were unaffected. The global isoenzymatic balance, even at high temperatures, suggests that these APX isozymes can keep the H_2_O_2_ content under control, including in extreme circumstances, and therefore this might be a mechanism to compensate the inhibition of some of the APX isozymes. The present data highlight the crosstalk between two signal molecules, NO and H_2_O_2_, during fruit ripening [89], a physiological process that is characterized by highly regulated nitro-oxidative stress [32], where NO seems to function upstream of ROS metabolism by differentially regulating the different CaAPX isozymes. The provided information could be useful for postharvest technology, since both molecules have proven to regulate the shelf life and nutritional quality of pepper fruits.

## Figures and Tables

**Table 1:** Sweet pepper genes encoding APX isozymes. Protein length as the number of amino acids (aa) corresponds to the pre-processed polypeptides. Theoretical pI and molecular weight (Mw) of each mature protein were predicted in silico using the Compute pI/Mw tool on the ExPASy server. Subcellular localization was predicted using WoLF PSORT.

Name	Gene	Nucleotide			Protein			**Localization**
		Chromosome Number	No. Introns	CDS Length (nt)	Length (aa)	Mw (kDa)	*pI*	
*CaAPX1*	LOC107859857	2	8	870	289	32.10	6.67	Peroxisome
*CaAPX2*	LOC107868078	4	12	1248	415	43.66	7.63	Plastid/ Mitochondrion
*CaAPX3*	LOC107873001	6	11	1038	345	37.84	8.67	Plastid/ Mitochondrion
*CaAPX4*	LOC107873435	6	9	753	294	32.45	5.85	Cytosol
*CaAPX5*	LOC107840564	8	8	864	287	31.61	7.10	Peroxisome
*CaAPX6*	LOC107842932	9	9	753	250	27.57	6.00	Cytosol

## Data Availability

Sequence Read Archive (SRA) data are available at the following link https://www.ncbi.nlm.nih.gov/sra/PRJNA668052 (accessed on 28 May 2020). On the other hand, the proteomic data were deposited in the repository PRIDE/ProteomeXchange with reference PXD010457.

## Supplementary Materials

The following supporting information can be downloaded at: https://www.mdpi.com/article/10.3390/antiox11040765/s1, Figure S1: Representative picture of the experimental strategy used in this study with the phenotype of sweet pepper (*Capsicum annuum* L.) fruits at different stages and treatments; Table S1: Percentage of amino acid identity among the six Ca-APX isozymes; Table S2. Evaluation of the best scored models of APX1 output by the servers M4T, Phyre2, Raptor X and Swiss model; Table S3: Evaluation of the best scored models of APX2 output by the servers M4T, Phyre2, Raptor X and Swiss model; Table S4: Evaluation of the best scored models of APX3 output by the servers M4T, Phyre2, Raptor X and Swiss model; Table S5. Evaluation of the best scored models of APX4 output by the servers M4T, Phyre2, Raptor X and Swiss model; Table S6. Evaluation of the best scored models of APX5 output by the servers M4T, Phyre2, Raptor X and Swiss model; Table S7. Evaluation of the best scored models of APX6 output by the servers M4T, Phyre2, Raptor X and Swiss model; Table S8. Main features of the models of the tertiary structure of the pepper APX isozymes; Table S9. Normalized RMS by alignment length and overall sequence length of the structural superposition of the models of the six APX isozymes; Table S10. Analysis of the superimposition of the six pepper APX isozymes on the structure of cytosolic pea APX (PDB entry 1APX).
