# Supplementary material for: Nitric Oxide (NO) Differentially Modulates the Ascorbate Peroxidase (APX) Isozymes of Sweet Pepper (Capsicum annuum L.) Fruits
Source: Antioxidants (Basel). 2022 Apr 12;11(4):765. doi: 10.3390/antiox11040765 (PMC9029456; doi:10.3390/antiox11040765)
Supplement: Supplementary file 1 [file antioxidants-11-00765-s001.zip › antioxidants-1673774-supplementary-done.pdf]

antioxidants

Figure S1. Representative picture of the experimental strategy used in this study with the phenotype of sweet pepper (*Capsicum annuum* L.) fruits at different stages and treatments: immature green, breaking point 1 (BP1), breaking point 2 without nitric oxide (NO) treatment (BP2 – NO), breaking point 2 with NO treatment (BP2 + NO), and ripe red. Pepper fruits were subjected to a NO-enriched atmosphere (5 ppm) in a methacrylate box for one hour and were then stored at room temperature (RT) for 3 days. Reproduced with permission from González-Gordo et al. (2020).

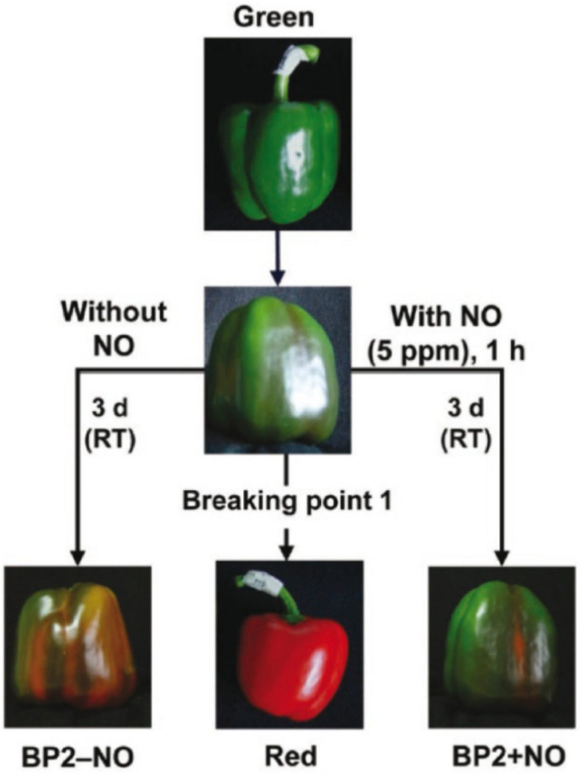

Table S1. Percentage of amino acid identity among the six Ca-APX isozymes.

**CaAPX1**	**100%**					
**CaAPX2**	50.61%	**100%**				
**CaAPX3**	51.84%	91.94%	**100%**			
**CaAPX4**	64.49%	45.56%	46.37%	**100%**		
**CaAPX5**	85.31%	49.80%	51.02%	55.75%	**100%**	
**CaAPX6**	66.12%	47.77%	48.58%	87.04%	67.35%	**100%**
	**CaAPX1**	**CaAPX2**	**CaAPX3**	**CaAPX4**	**CaAPX5**	**CaAPX6**

Table S2. Evaluation of the best scored models of **APX1** output by the servers M4T, Phyre2, Raptor X and Swiss model

Sever	model	Qmean4[a] (Qmean-Z score[b])	Errat[c]	Verify3D[d]	Procheck[e]
**M4T**	1	0.782 (0.101)	90.3766	86.64%	95.7% (0.5%)
**Phyre2**	1	0.785 (0.119)	86.6953	98.77%	95.6% (0.5%)
	2	0.790 (0.284)	88.0851	100%	96.6% (0.5%)
	3	0.669 (-2.634)	79.5745	99.59%	87.5% (0.5%)
	4	0.663 (-2.711)	69.6581	76.31%	90.4% (0.5%)
	5	0.590 (-4.514)	56.1983	84.22%	78.9% (1.4%)
	8	0.661 (-2.759)	82.6613	91.60%	90.6% (1.3%)
	11	0.729 (-1.178)	82.8326	90.12%	90.8% (0.0%)
	14	0.557 (-5.364)	58.4677	81.64%	87.0% (0.9%)
	19	0.534 (-5.899)	58.1673	74.52%	86.9% (0.9%)
**RaptorX**	1	0.676 (-2.436)	72.1569	73.01%	92.8% (0.4%)
**Swiss Model**	1	0.799 (0.515)	98.7342	100%	94.7% (0.0%)

[a] Model reliability ranging from 0 to 1.

[b] *Degree of nativeness*. QMEAN Z-score directly indicates how many standard deviations the model's QMEAN score differs from expected values for experimental structures.

[c] The error function is based on the statistics of non-bonded atom-atom interactions in the reported structure (compared to a database of reliable high-resolution structures). Generally speaking, the method is sensitive to smaller errors than 3-D Profile analysis.

[d] Analyzes the compatibility of an atomic model (3D) with its own amino acid sequence (1D). Each residue is assigned a structural class based on its location and environment (alpha, beta, loop, polar, nonpolar, etc.).

[e] Percentage of residues in the most favored regions of the Ramachandarn plot. In bracket those in the disallowed regions.

Table S3. Evaluation of the best scored models of **APX2** output by the servers M4T, Phyre2, Raptor X and Swiss model

Sever	model	Qmean4[a] (Qmean-Z score[b])	Errat[c]	Verify3D[d]	Procheck[e]
**M4T**	1	0.770 (-0.092)	91.3858	100%	96.5% (0.0%)
**Phyre2**	1	0.799 (0.632)	92.2406	97.45%	92.4% (0.0%)
	2	0.582 (-4.790)	61.4545	75.79%	89.6% (0.4%)
	3	0.579 (-4.834)	62.3616	78.80%	87.8% (1.3%)
	4	0.587 (-4.679)	66.6667	78.87%	90.8% (0.8%)
	5	0.526 (-6.617)	56.25	80.94%	79.0% (1.2%)
	7	0.519 (-6.337)	67.5	78.60%	87.3% (0.9%)
	16	0.466 (-8.300)	38.0952	77.15%	83.7% (1.2%)
**RaptorX**	1	0.678 (-2.739)	76.1146	85.00%	90.1% (1.8%)
**Swiss Model**	1	0.792 (0.457)	93.633	100%	94.8% (0.0%)

[a] Model reliability ranging from 0 to 1.

[b] *Degree of nativeness*. QMEAN Z-score directly indicates how many standard deviations the model's QMEAN score differs from expected values for experimental structures.

[c] The error function is based on the statistics of non-bonded atom-atom interactions in the reported structure (compared to a database of reliable high-resolution structures). Generally speaking, the method is sensitive to smaller errors than 3-D Profile analysis.

[d] Analyzes the compatibility of an atomic model (3D) with its own amino acid sequence (1D). Each residue is assigned a structural class based on its location and environment (alpha, beta, loop, polar, nonpolar, etc.).

[e] Percentage of residues in the most favored regions of the Ramachandarn plot. In bracket those in the disallowed regions.

Table S4. Evaluation of the best scored models of **APX3** output by the servers M4T, Phyre2, Raptor X and Swiss model

Sever	model	Qmean4[a] (Qmean-Z score[b])	Errat[c]	Verify3D[d]	Procheck[e]
**M4T**	1	0.776 (0.042)	90.566	95.60%	95.6% (0.0%)
**Phyre2**	1	0.793 (0.496)	95.8491	100%	92.4% (0.0%)
	2	0.566 (-5.097)	53.1381	82.47%	87.0% (0.5%)
	3	0.503 (-6.698)	58.6066	77.95%	82.2% (1.0%)
	4	0.567 (-5.145)	67.2065	87.45%	91.8% (0.5%)
	5	0.551 (-5.537)	69.962	76.57%	89.0% (0.4%)
	6	0.604 (-3.737)	75.2294	91.63%	83.5% (0.0%)
	7	0.571 (-4.972)	68.3128	86.17%	90.4% (0.5%)
	15	0.549 (-5.874)	73.7226	81.36%	90.0% (0.4%)
**RaptorX**	1	0.751 (-0.569)	92.3077	95.04%	94.0% (0.9%)
**Swiss Model**	1	0.789 (0.374)	93.5849	100%	94.7% (0.0%)

[a] Model reliability ranging from 0 to 1.

[b] *Degree of nativeness*. QMEAN Z-score directly indicates how many standard deviations the model's QMEAN score differs from expected values for experimental structures.

[c] The error function is based on the statistics of non-bonded atom-atom interactions in the reported structure (compared to a database of reliable high-resolution structures). Generally speaking, the method is sensitive to smaller errors than 3-D Profile analysis.

[d] Analyzes the compatibility of an atomic model (3D) with its own amino acid sequence (1D). Each residue is assigned a structural class based on its location and environment (alpha, beta, loop, polar, nonpolar, etc.).

[e] Percentage of residues in the most favored regions of the Ramachandarn plot. In bracket those in the disallowed regions.

Table S5. Evaluation of the best scored models of **APX4** output by the servers M4T, Phyre2, Raptor X and Swiss model

Sever	model	Qmean4[a] (Qmean-Z score[b])	Errat[c]	Verify3D[d]	Procheck[e]
**M4T**	1	0.800 (0.557)	91.7012	100%	95.7% (0.0%)
**Phyre2**	**1**	0.827 (1.203)	95.7	100%	94.2% (0.5%)
	**2**	0.586 (-4.646)	67.9167	82.56%	82.1% (0.5%)
	**3**	0.789 (0.275)	97.9253	93.39%	94.2% (0.5%)
	**4**	0.532 (-6.005)	44.1767	87.16%	84.6% (1.4%)
	**5**	0.660 (-2.881)	65.4867	100%	89.3% (0.0%)
	**6**	0.596 (-4.394)	61.2	81.61%	87.9% (0.5%)
	**7**	0.523 (-6.130)	57.6132	84.46%	84.7% (1.0%)
	**9**	0.514 (-6.446)	40	87.94%	75.6% (0.0%)
	**20**	0.669 (-2.614)	67.364	82.59%	87.3% (0.5%)
**RaptorX**	1	0.694 (-2.082)	70.2206	82.99%	93.5% (0.0%)
**Swiss Model**	1	0.814 (0.897)	96.2343	99.19%	95.1% (0.0%)

[a] Model reliability ranging from 0 to 1.

[b] *Degree of nativeness*. QMEAN Z-score directly indicates how many standard deviations the model's QMEAN score differs from expected values for experimental structures.

[c] The error function is based on the statistics of non-bonded atom-atom interactions in the reported structure (compared to a database of reliable high-resolution structures). Generally speaking, the method is sensitive to smaller errors than 3-D Profile analysis.

[d] Analyzes the compatibility of an atomic model (3D) with its own amino acid sequence (1D). Each residue is assigned a structural class based on its location and environment (alpha, beta, loop, polar, nonpolar, etc.).

[e] Percentage of residues in the most favored regions of the Ramachandarn plot. In bracket those in the disallowed regions.

Table S6. Evaluation of the best scored models of **APX5** output by the servers M4T, Phyre2, Raptor X and Swiss model

Sever	model	Qmean4[a] Qmean-Z score[b]	Errat[C]	Verify3D[d]	Procheck[e]
**M4T**	1	0.741 (-0.855)	84	95.35%	94.5% (0.5%)
**Phyre2**	1	0.717 (0.805)	83.691	99.59%	96.6% (0.0)
	2	0.548 (0.805)	59.0164	83.20%	86.6% (1.8%)
	3	0.564 (-5.141)	56.9721	81.92%	88.7% (0.9%)
	4	0.544 (-5.687)	58.4677	81.64%	85.3% (1.4%)
	5	0.645 (-3.211)	72.4576	97.13%	86.5% (0.0%)
	6	0.806 (0.689)	90.2128	97.53%	97.1% (0.0%)
	18	0.715 (-1.547)	87.931	91.36%	91.3% (0.5%)
**RaptorX**	1	0.681 (-2.310)	74.2308	77.70%	92.3% (1.2%)
**Swiss Model**	1	0.812 (0.870)	97.4684	100%	96.7% (0.0%)

[a] Model reliability ranging from 0 to 1.

[b] *Degree of nativeness*. QMEAN Z-score directly indicates how many standard deviations the model's QMEAN score differs from expected values for experimental structures.

[c] The error function is based on the statistics of non-bonded atom-atom interactions in the reported structure (compared to a database of reliable high-resolution structures). Generally speaking, the method is sensitive to smaller errors than 3-D Profile analysis.

[d] Analyzes the compatibility of an atomic model (3D) with its own amino acid sequence (1D). Each residue is assigned a structural class based on its location and environment (alpha, beta, loop, polar, nonpolar, etc.).

[e] Percentage of residues in the most favored regions of the Ramachandarn plot. In bracket those in the disallowed regions.

Table S7. Evaluation of the best scored models of **APX6** output by the servers M4T, Phyre2, Raptor X and Swiss model

Sever	model	Qmean4[a] (Qmean-Z score[b])	Errat[C]	Verify3D[d]	Procheck[e]
**M4T**	1	0.818 (0.982)	86.25	99.60%	95.1% (0.0%)
**Phyre2**	1	0.838 (1.451)	88.2845	100%	95.6% (0.0%)
	2	0.823 (1.100)	95.8159	100%	95.6% (0.0%)
	3	0.549 (-5.525)	43.5895	95.45%	81.9% (1.5%)
	7	0.669 (-2.678)	77.9736	94.09%	92.2% (0.5%)
	10	0.708 (-1.656)	69.6203	93.95%	90.8% (0.5%)
	11	0.627 (-3.706)	68.3036	90.30%	85.7% (2.0%)
**RaptorX**	1	0.810 (0.800)	88.0165	98.40%	96.6% (0.0%)
**Swiss Model**	1	0.851 (1.773)	97.8814	100%	96.6% (0.0%)

[a] Model reliability ranging from 0 to 1.

[b] *Degree of nativeness*. QMEAN Z-score directly indicates how many standard deviations the model's QMEAN score differs from expected values for experimental structures.

[c] The error function is based on the statistics of non-bonded atom-atom interactions in the reported structure (compared to a database of reliable high-resolution structures). Generally speaking, the method is sensitive to smaller errors than 3-D Profile analysis.

[d] Analyzes the compatibility of an atomic model (3D) with its own amino acid sequence (1D). Each residue is assigned a structural class based on its location and environment (alpha, beta, loop, polar, nonpolar, etc.).

[e] Percentage of residues in the most favored regions of the Ramachandarn plot. In bracket those in the disallowed regions.

Table S8. Main features of the models of the tertiary structure of the pepper APX isozymes

Isoform	Template[1]	% Identity[2]	Range (coverage)[3]	Server
Ca-APX1	2ghh	62.45	2-246 (85%)	Swiss model
Ca-APX2	1iyn	86.05	90-364 (71%)	Swiss model
Ca-APX3	1iyn	90.84	73-345 (78%)	Phyre2
Ca-APX4	2vcs	82.00	46-293 (85%)	Swiss model
Ca-APX5	2ghh	62.45	2-246 (85%)	Swiss model
Ca-APX6	2y6a	83.87	2-248 (99%)	Swiss model

[1] Templated used for the modeling: **2ghh**: *Glycine max* APX (cytosolic), **1iyn**: *Nicotiana tabacum* APX (chloroplastic), **2vcs**: *Glycine max* APX (cytosolic), **2y6a** *Glycine max* APX (R38A mutant).

[2] Identity between the modeled sequence and the template

[3] Residues (in brackets the percentage) modeled.

Table S9. Normalized RMS by alignment length and overall sequence length of the structural superposition of the models of the six APX isozymes.

**CaAPX1**	**0**					
**CaAPX2**	0.970	**0**				
**CaAPX3**	0.970	0.260	**0**			
**CaAPX4**	0.226	0.975	0.975	**0**		
**CaAPX5**	<u>0.073</u>	0.969	0.969	0.231	**0**	
**CaAPX6**	0.250	0.983	0.983	0.252	0.256	**0**
	**CaAPX1**	**CaAPX2**	**CaAPX3**	**CaAPX4**	**CaAPX5**	**CaAPX6**

Table S10. Analysis of the superimposition of the six pepper APX isozymes on the structure of cytosolic pea APX (PDB entry 1APX)

	**CaAPX1**	**CaAPX2**	**CaAPX3**	**CaAPX4**	**CaAPX5**	**CaAPX6**
**Rmsd[1]**	0.431	0.980	0.979	0.335	0.436	0.317
**SDM[2]**	8.609	19.616	19.606	6.704	8.716	6.556
**Q-score[3]**	0.964	0.735	0.740	0.976	0.964	0.973

[1] Normalized RMSD by alignment length and overall sequence length

[2] Structural Distance Measure (SDM) is zero for identical structures and increases as the similarity decreases.

[3] Q-score range from zero for completely dissimilar or un-superimposed structures to 1 for identical structures.
